# Implementation of Established Conditions and Use of Quality by Design Principles during Drug Development: Status in the US, EU, and Japan. Data from a Survey Conducted by the Japan Pharmaceutical Manufacturers Association (JPMA)

**DOI:** 10.1007/s43441-025-00856-1

**Published:** 2025-08-21

**Authors:** Yoshio Nakayama, Sonoko Yamauchi, Kozue Shimizume, Akinobu Nakanishi, Maki Masuyama, Yasuyo Ozaki, Koji Nakamura, Makoto Fujikawa, Masatsugu Kobayashi, Yuji Kashitani

**Affiliations:** 1 Eli Lilly Japan K.K, 5-1-28 Isogami-dori, Chuo-ku, Kobe, 651-0086 Japan; 2 Kyowa Kirin Co., Ltd, Tokyo, Japan; 3 Meiji Seika Pharma Co., Ltd, Tokyo, Japan; 4 TEIJIN PHARMA LIMITED, Tokyo, Japan; 5 MSD K.K, Tokyo, Japan; 6 AstraZeneca K.K, Osaka, Japan; 7 Torii Pharmaceutical Co., Ltd, Tokyo, Japan; 8 DAIICHI SANKYO COMPANY, LIMITED, Tokyo, Japan; 9 Sumitomo Pharma Co., Ltd., Osaka, Japan; 10 Takeda Pharmaceutical Company Limited, Osaka, Japan; 11 JPMA Regulatory Affairs Committee, General Regulatory Sub-Committee, Tokyo, Japan; 12 JPMA Regulatory Affairs Committee, Tokyo, Japan

**Keywords:** Post approval changes (PACs), Established conditions (ECs), ICH Q12, Quality by design (QbD), PQS (Pharmaceutical quality systems)

## Abstract

The JPMA conducted a survey among its member companies regarding the use of Established Conditions (ECs) under ICH Q12. ECs can be set by companies that develop new drugs using the Quality by Design (QbD) approach defined in ICH Q8 and have an effective Pharmaceutical Quality System (PQS) as per ICH Q10. The survey revealed that while the use of QbD has increased, surpassing 70% in Japan since 2021, the adoption of ECs in New Drug Application (NDA) submissions remains low due to a lack of legal framework and internal understanding. More companies were using ECs in post-approval changes (PACs) compared to NDA submissions. The survey also found that companies prefer the existing systems in each region when determining the change category during change initiation. While Europe and the US believe that risk assessment of changes and ECs are consistent with an effective PQS, Japan perceives a mismatch between change assessment and predetermined change categories at the time of approval. This results in Japan willing to have an option applying the risk assessment at change control to reporting category evaluation. Considering these circumstances, it is anticipated that the use of ECs will gradually expand, primarily in PACs. The discrepancies in change procedures among countries may hinder a stable supply, so Japan should consider introducing change guidelines similar to those in Europe and the US to facilitate a hybrid approach to approvals that can accommodate the expanded use of ECs.

## Introduction

ICH guidelines, Q8 [1], Q9 [2], and Q10 [3] are often referred to as the Q Trio Guidelines (after adding Q11 [4], it became the Q Quartet). These guidelines were established by ICH to: “*Develop a harmonised pharmaceutical quality system applicable across the life cycle of the product emphasizing an integrated approach to quality risk management and science”.* This vision was intended to ensure quality products and reliable supply to patients. ICH developed these guidelines [5] to ensure product quality and stable supply. Proper interpretation and use of these guidelines makes it possible to systematically implement a risk-based approach to drug quality assurance, change management, and drug development.

The use of Quality by Design (QbD) principles as established by ICH Q8 was initially limited [6, 7] and expanded when the ICH Points to Consider document for the implementation of ICH Q8/Q9/Q10 [8] was published [9, 10]. Elements of QbD have been used for several years in the EU [11].

The ICH Q12 Guideline; Technical and Regulatory Considerations for Pharmaceutical Product Lifecycle Management [12] and its Annexes: Technical and Regulatory Considerations for Pharmaceutical Product Lifecycle Management [13] reached Step 4 in 2019, with the expectation that a harmonized post-approval assessment and submission process would lead to review efficiency and the ability to implement changes simultaneously in multiple markets. As of December 2024, only 3 countries have fully implemented Q12; 9 are preparing to implement, and 4 have not yet started.

Among the tools provided in Q12, Established Conditions (ECs) and the Product Lifecycle Management document (PLCM) can give flexibility in Post-Approval Changes (PACs) when developed by a company with an appropriate product quality system as per Q10 [12]. Although QbD may not decrease the review time for products using this approach, the use of QbD has increased in Japan, where 71% of applications used this approach in 2019 [10]. For this reason, it seems that many products are ready to use Q12 tools, and it is possible to set ECs. These of QbD principles took several years before being submitted for review regularly and the situation may be similar for regular use of ECs.

In order to clarify the actual implementation status of Q12 ECs in the ICH original member regions of Japan, the United States, and Europe, we developed a survey that JPMA member companies and their overseas parent companies, and their affiliates, provided information regarding the number of applications that included ECs from the time Q12 reached Step 4 in 2019 until April 2024 as well as reasons for not using ECs, and if there is a plan to define them in the future. The survey also asked about preference of review timing for change category assessment and explored perceived obstacles to the use of ECs and the PLCM.

## Strategy and Method

We asked JPMA member companies to answer the questions in cooperation with their overseas parent companies and their affiliates regarding whether they had experience in setting and applying ECs under ICH Q12 for approval in Japan, the U.S. and Europe, and included questions about the risk assessment of changes.

For the survey, an Excel questionnaire was sent to 66 companies (company names were not tracked). Responses were received from 60 companies (42 domestic to Japan, 17 foreign affiliated companies and 1 did not respond to the question on location). In addition to typical responses “cannot answer” was also an option.

## Results

### ICH Q12 Established Conditions Implementation in the US, EU and Japan

From November 20, 2019, when Q12 reached Step 4, to the time of the survey, we asked about the number of new drug applications submitted to Japan, the United States, and Europe, including the number of applications with ECs, and, if applicable, the reasons for not setting ECs. We examined the experience of setting ECs at the time of PACs and investigated plans for defining ECs in future applications.

**Fig. 1:**
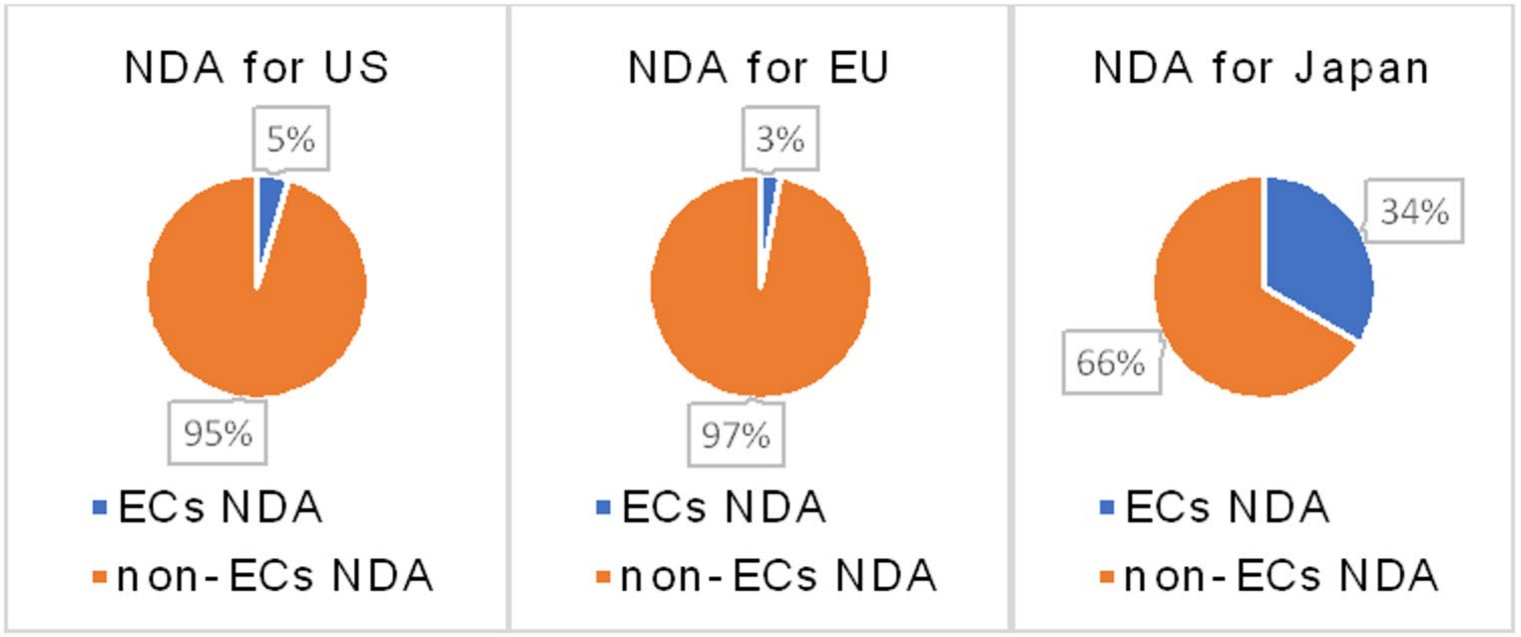
ECs or non-ECs including NDAs by regions (submission number bases)

**Fig. 2:**
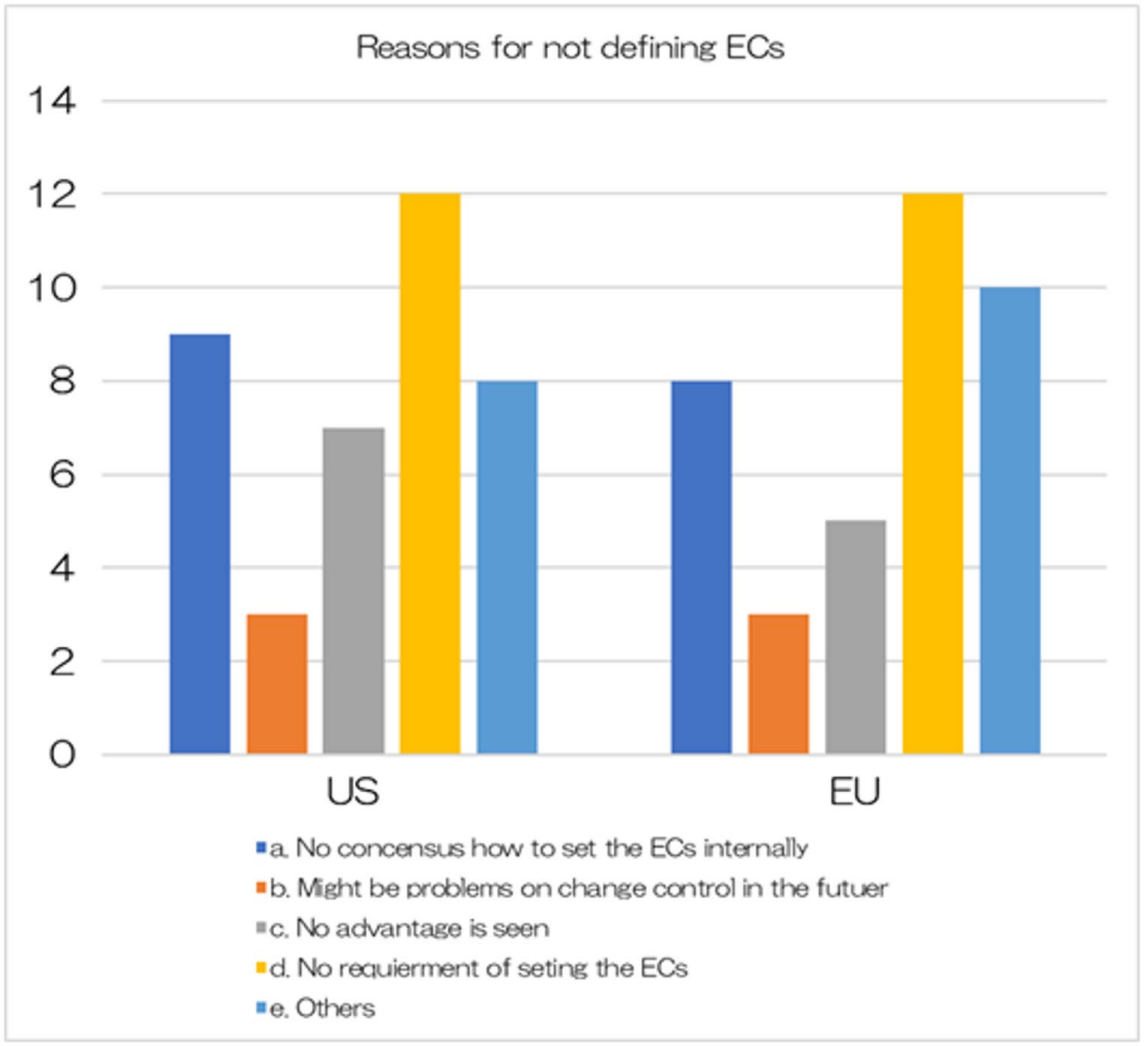
Reasons for not defining ECs

As shown in Fig. 1, there were very few applications in Europe and the United States that defined ECs. During that period, only 4 of the 21 companies (19%) that had submitted new drug applications to the U.S. had defined ECs. Of the 20 companies that submitted new drug applications in Europe during that period, only 1 company had defined ECs (5%). Additionally, none of the applications made by Japanese domestic companies to Europe or the United States defined ECs. Details are shown in Table 1. As shown in Fig. 2, the most common reason for not defining ECs in both Europe and the U.S. was that it is not a requirement to do so. Other reasons included a lack of understanding of ECs within the company, and not perceiving the benefits of defining ECs. There were also many responses that selected “Other,” with reasons such as ECs being limited to pilot cases in the U.S. and there remains to be no legal basis in Europe for their use. Some respondents who did not define ECs were concerned about a review delay because using ECs is a new concept or that they were unclear about the potential benefit. Comment from respondents answering not defined ECs at New Drug Application (NDA), they mentioned that they felt unclear benefit on ECs and thought it might be a possibility of review delay.

**Fig. 3:**
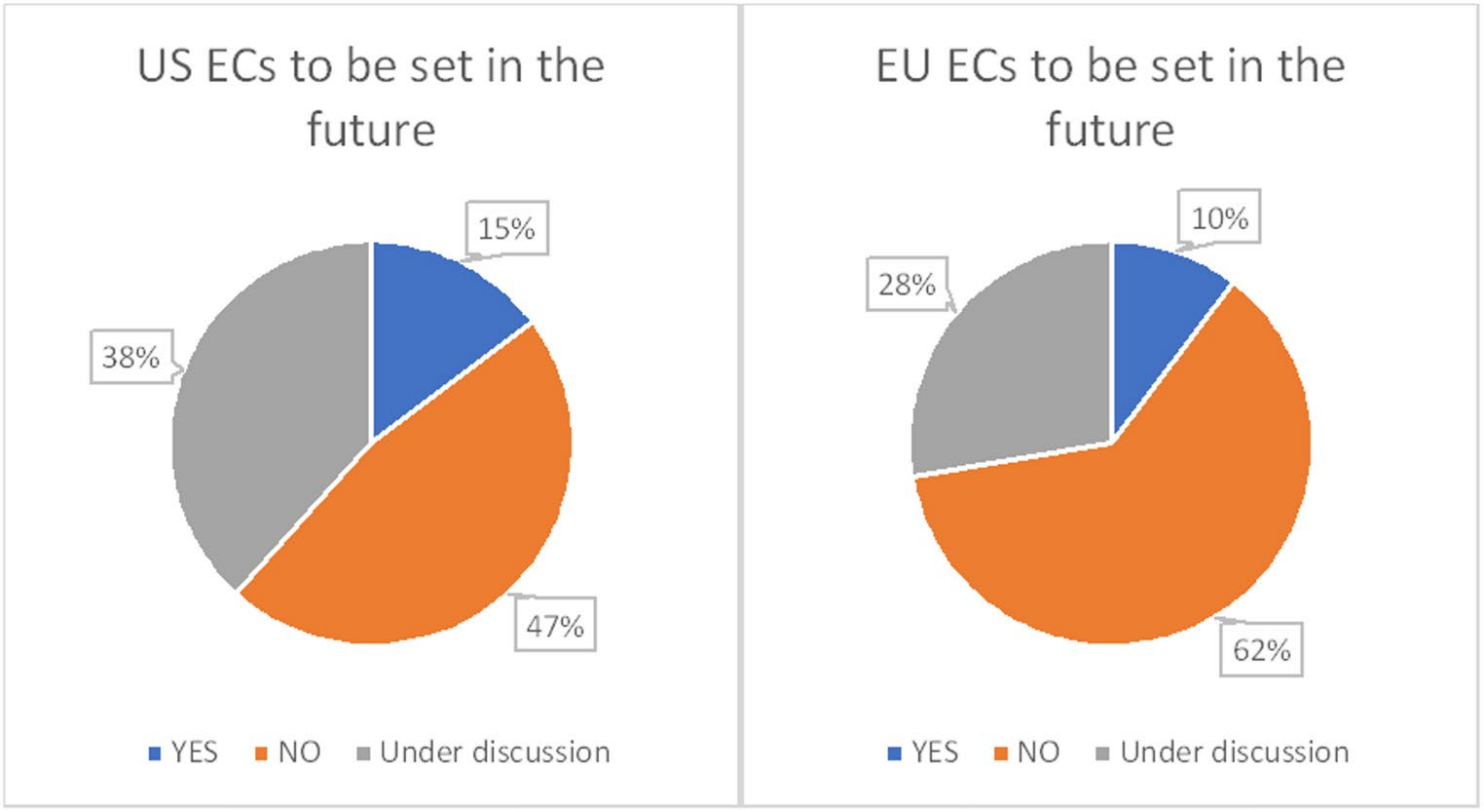
Will ECs be defined in the future for products the US and EU?

As shown in Tables 2 and 7 companies submitted 19 post-approval submissions to the US FDA in which they defined ECs. Similarly, there were 14 post-approval submissions to the EU from 1 company in which ECs were defined. All of the responses were from foreign-affiliated companies (Japanese affiliate of the global companies). As shown in Figs. 3 and 5 out of 34 respondents (15%) have plans to define ECs in their applications to the U.S., 16 (47%) have no plans, and 13 (38%) are considering their use. In addition, 3 out of 29 companies (10%) responded that they plan to apply to Europe, 18 (62%) have no plans, and 8 (28%) are under consideration.

On the other hand, as shown in Fig. 1; Tables 1 and 11 (28%) of the 39 companies (28 domestic, 10 foreign, and 1 non-respondent) that had applied to Japan during the same time period had defined ECs. Of the 191 applications, 64 (34%) were submitted with ECs.

**Fig. 4:**
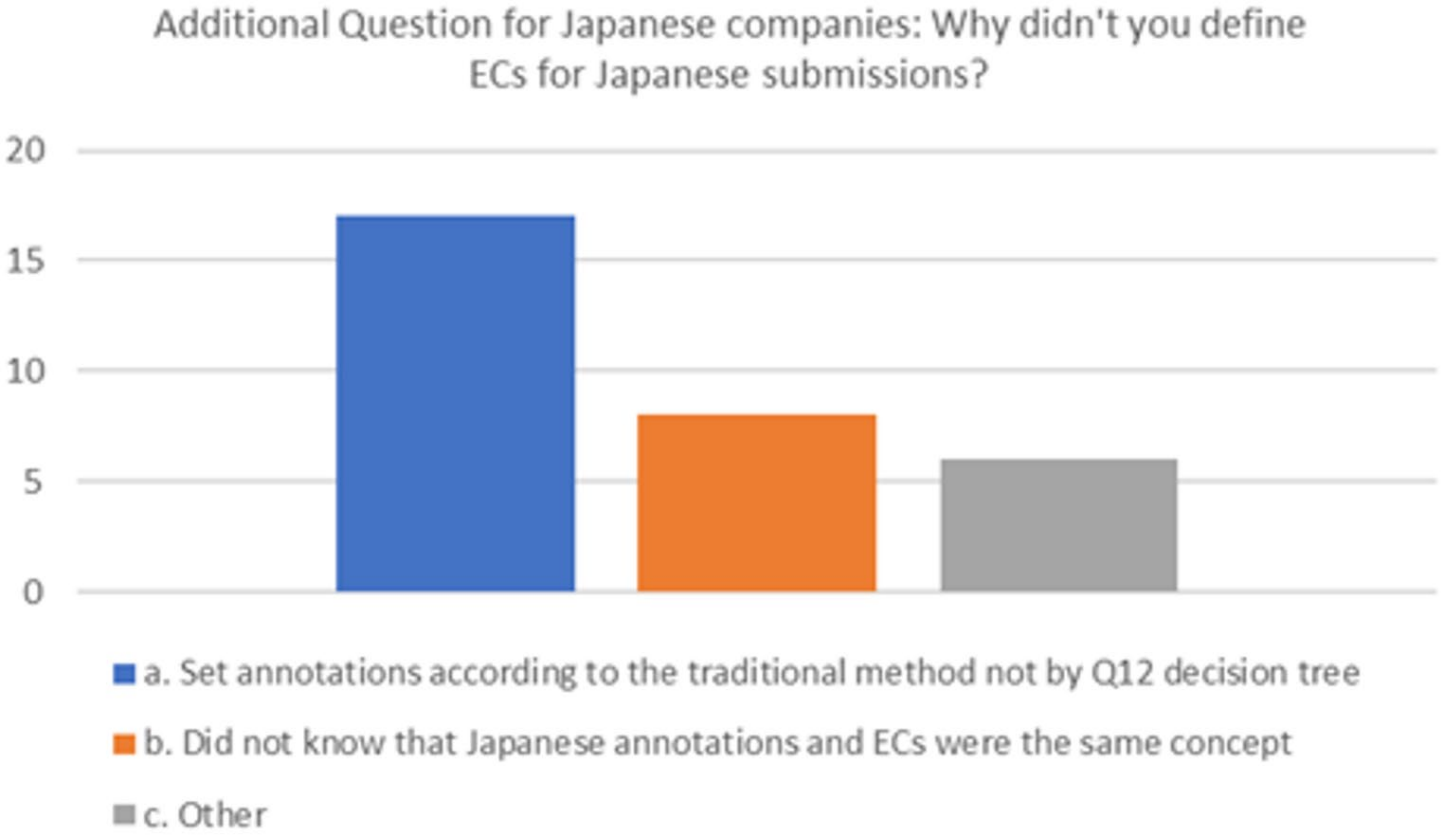
Additional question to Japanese companies regarding why they did not define ECs in NDA submissions to Japan (Multiple answers allowed)

Of the companies that submitted in Japan without defining ECs, Fig. 4 shows that, of the total 31 responses, 17 (55%) did not assess the impact on quality or consider the risks of each parameter in accordance with the decision tree shown in ICH Q12, and 8 (26%) thought that the symbols for changes or minor changes in EC and Japan in ICH Q12 were different. There were also six that responded “other” (19%).

**Table 1:** Number of NDAs submitted between 2019 to 2024

	Total	ECs NDA	non-ECs NDA
TOTAL NDAs	383*	72	312
US NDAs	85*	5	81
EU NDAs	107	3	104
JP NDAs	191	64	127
Japanese companies NDAs	159	24	135
Foreign companies NDAs	219*	48	172
Unknown companies NDAs	5	0	5
Japanese companies US NDAs	22	0	22
Japanese companies EU NDAs	30	0	30
Japanese companies JP NDAs	107	24	83
Unknown companies US NDAs	1	0	0
Unknown companies EU NDAs	1	0	0
Unknown companies JP NDAs	3	0	0
Foreign companies US NDAs	62*	5	58
Foreign companies EU NDAs	76	3	73
Foreign companies JP NDAs	81	40	41

*There is a disconnect in the total count due to inconsistent responses received. However, the total number of submissions in the table was used for calculations

**Table 2:** ECs setting by PACs

Submission to	Yes (Companies (application numbers))	No (Companies)
US	7 (19)	27
EU	1 (14)	29

### Assessing a Planned Post-Approval Change

Japan’s CMC PAC system relies on symbols, or annotations, that define which reporting category will be used in the future when changes are made (partial change application or minor change notification) and are finalized at approval of the NDA [14]. This process was implemented in 2005. The ICH Q Quartet was issued from 2006 to 2009 and Q12 reached Step 4 in 2021, however, these guidelines were published after the Japanese manufacturing process description and change system process introduction. The ECs in Q12 also indicate the category of changes to be used when the parameters selected as ECs are changed in the future [12] and this is the same as result as using the traditional annotations in Japan [14]. Unlike the United States and Europe, Japan does not have additional guidance explaining what type of post-approval submission is needed as this should be clearly defined during the review process [16–19]. The US and EU allow for a risk-based approach to the reporting category dependent on the impact to product quality, safety, and efficacy.

**Fig. 5:**
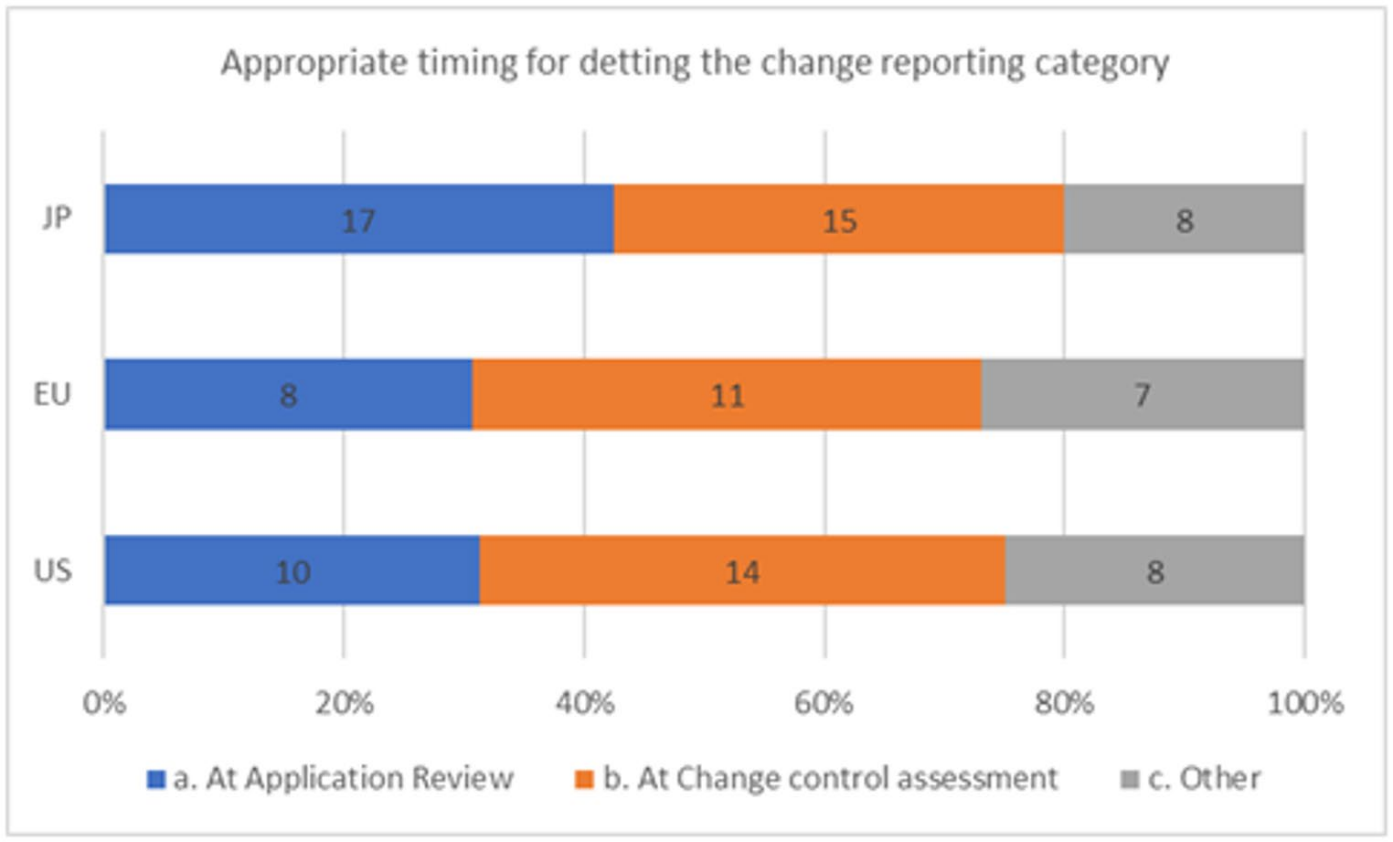
The most appropriate timing to decide the change category setting by regions

When asked via the survey when is the most appropriate time to define the regulatory reporting category, Fig. 5 shows that, in the U.S., 10 (31%) thought it would be best to define the reporting category during application review, 14 (44%) said at the time the change was planned, and 8 responded “other” (25%). For Europe, 8 (31%) responded at new drug application review, 11 (42%) was at the time the change was being planned and 7 (27%) said “other”. Finally for Japan, 17 (43%) responded at new drug application review, 15 (38%) said at the time the change was being planned and 8 (20%) responded “other.” There is a tendency to think that it would be more appropriate to use the existing regulatory change reporting system currently in place in each region.

**Fig. 6:**
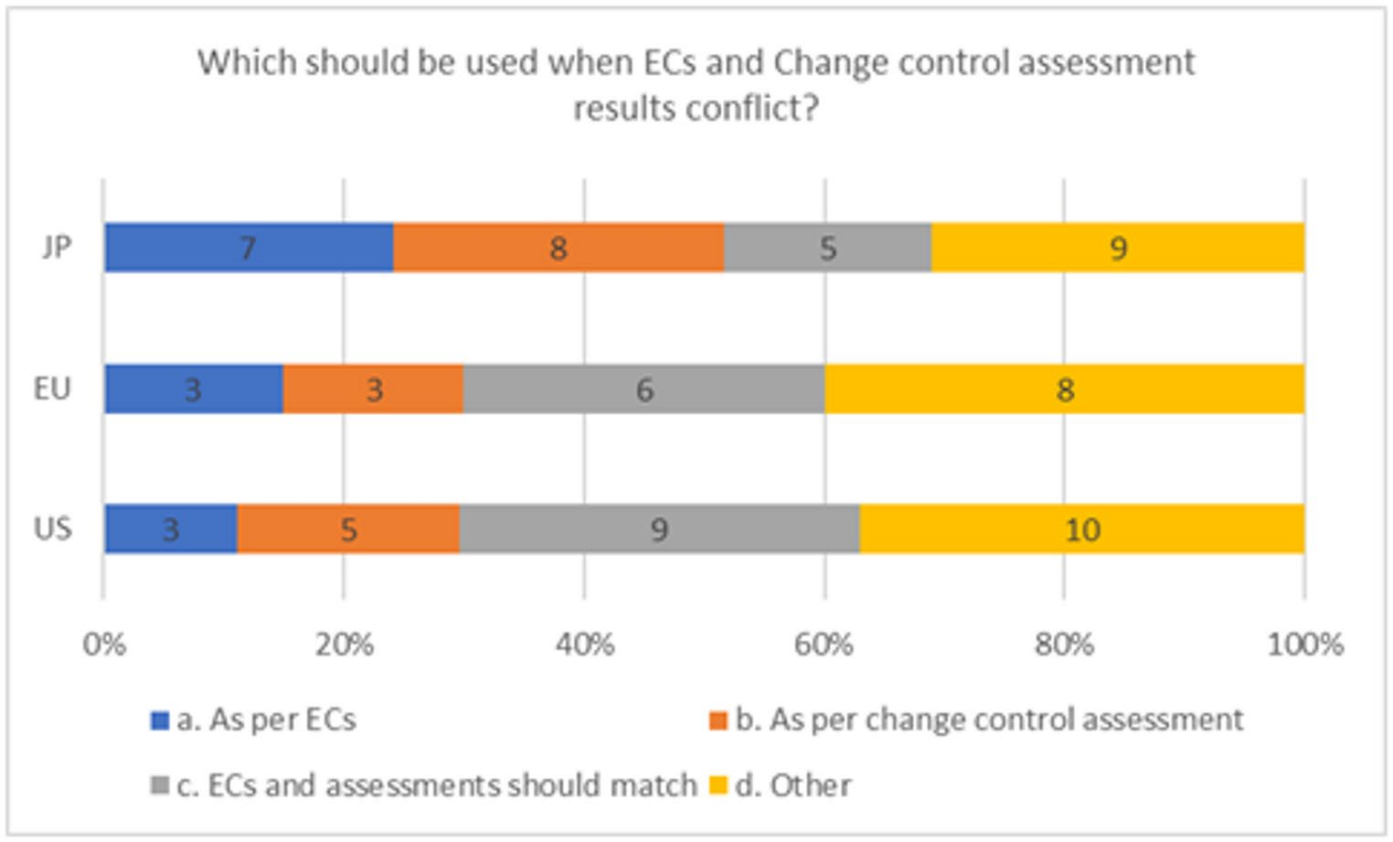
Respected conditions when there is a conflict between ECs and change assessment for a change

We asked if companies already defined ECs in the commitments, and there were discrepancies between the regulatory reporting category for ECs and risk assessment outcome from GMP change control, which one should be followed? As shown in Fig. 6, in the U.S., 3 (11%) of sponsors answered that the change category as defined by ECs should be followed; 5 (19%) said that the risk assessment should be followed; 9(33%) said the ECs and change controls assessment should match; and 10 (37%) indicated “other”. European respondents answered: 3(15%) that ECs should be followed; 3 (15%) indicated that the change control assessment should be followed; 6 (30%) stated that the two should match; and 8 (40%) responded “other”. Japan-based respondents indicated 17 (24%) should follow the ECs; 8 (28%) that the change controls assessment should be followed; 5 (17%) stated that the two should match; and 9 (31%) stated “other”. In all cases, the major answer was “other” in those cases where respondents added additional information for that category the common theme was that they would negotiate with the health authorities where there was a conflict between the ECs and the change control assessment. In Japan, one respondent said it would be done on a case-by-case basis and the others indicated that they did not have ECs in place or had no experience with this situation and could not provide more information.

### Status of Quality by Design Use during Development

ICH Q12 tools are subject to proper implementation of Q8 and Q10. We determined the number of new chemical entities, including biosimilars, that were approved in Japan using QbD principles. These data were obtained from PMDA review reports between 2019 when Q12 reached Step 4 and 2023. Specifically, when terms such as QbD, CQA, Critical Quality Attributes, Control Strategies, Quality Risk Management, and Design Space were seen in the reports the product was assessed as being developed using QbD. The Q12 guideline states that the extent of ECs may vary based on the company’s development approach, product and process understanding, and the potential risk to product quality, however, there is no clear indicator of how these expectations are met. Therefore, the use of QbD was used as a surrogate to indicate products that could define a range of ECs.

**Fig. 7:**
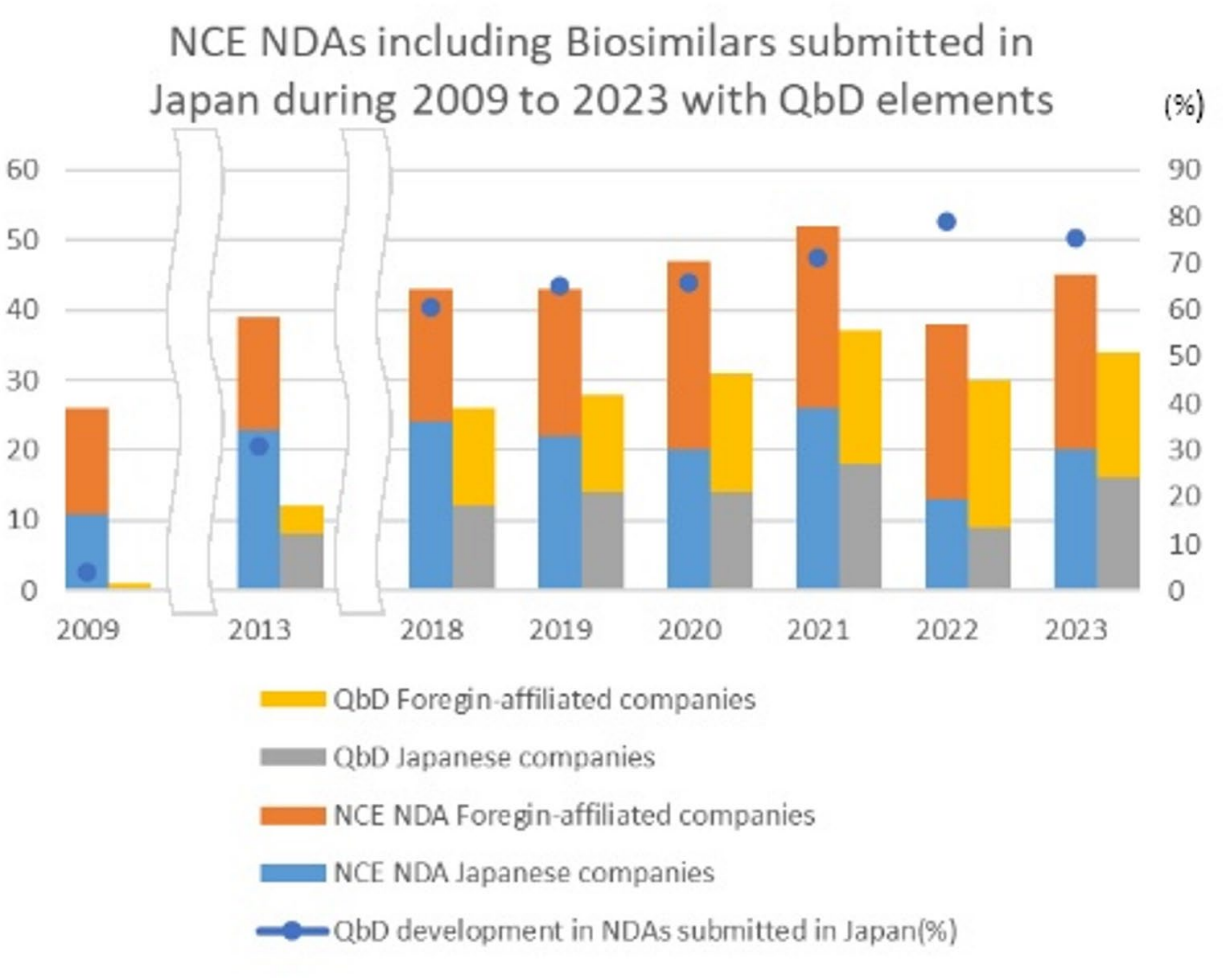
New drugs approved in Japan using elements of QbD

As shown in Fig. 7, it was confirmed that new drug approvals using QbD during development increased over time by more than 60% since 2018, 65% in 2019 when ICH Q12 reached Step 4, and more than 70% since 2021. This trend could only be investigated for products for which PMDA review reports were published, and it was not possible to identify whether QbD was used in applications for partial change applications or generic products.

## Discussion

Although there appears to be a clear increase in the use of QbD principles during the drug development process (Fig. 7), defining ECs in regulatory submissions remains limited in the US and EU (Fig. 1). Therefore, we examined the potential reasons for the slow adoption of ECs.

The United States and Europe have had post approval change guidelines in place since 1999 and 2008, respectively and they are still in use today.

ICH Q8/9/10 guidelines reached Step 4 in 2006, followed by a revision for Q8 in 2010 and for Q9 in 2023. There was slow adoption of QbD principles due to a lack of understanding of how best to use this approach, and the amount of work that needs to be done during development when it is unclear as to whether or not the product will be successful. To assist in their use, ICH published a Points to Consider document for Q8/9/10 in 2011 which was revised in 2013.

In Europe, it was reported that only 38% of new drug applications were developed using full QbD between 2014 and 2019. Several products were not developed via full QbD implementation but instead used one or more QbD elements, including design space [11]. The use of QbD development in Japan marketing applications began in 2009 when the description that: it was developed by QbD elements or quality risk assessment” appeared in PMDA new drugs review reports (including biosimilars). As shown in Fig. 7, in 2009, 4% of marketing applications used elements of QbD (including 1 item/26 items, including 2 biosimilars: however, the application was made using the experimental design method), in 2013 it was 31% (12 items/39 items), 60% by 2018 (26/43 items, including biosimilars), and in 2021, when Q12 reached Step 4, it was 71% (37/52 items, including biosimilars), in 2022, it was 79% (30 items/38 items), and in 2023, it was 76% (34 items/45 items). In recent years it has stabilized at more than 70%. Not all new drugs approved in Japan are approved in the EU and the US, still, the data show that the QbD approach is being used more often in the development of products.

The Q12 guideline preparation discussion began in 2014 and reached Step 4 in 2021. “This guideline addresses the commercial phase of the product lifecycle (as described in ICH Q10); and it both complements and adds to flexible regulatory approaches to post-approval CMC changes described in ICH Q8(R2) and Q10 Annex 1.“ [12]. It allows companies that effectively operate the PQS, as indicated in Q10, to use various Q12 tools, including ECs, if they develop products using the methods outlined in Q8 and Q11 and perform appropriate risk assessments according to Q9. Tools and enablers described in Q12 are complementary and are intended to link different phases of the product lifecycle [12].

The number of products approved using the QbD approach has been increasing, so it is believed that many products can set ECs, which is a tool available in Q12. However, as of 2024, ECs are still rarely used in the EU and the US even when QbD principle as are used during development of a product. Note that at the time of the survey, the legal framework for accepting ECs was not in place in the EU and therefore sponsors could not submit applications defining ECs even if they had the supporting data available.

Figure 2 shows that the primary reason for not setting ECs is that companies generally remain reluctant to do so because of the uncertainty it adds with regards to approval of a new drug application. Not receiving approval would have impact beyond manufacturing and testing to patient access and business-related predictions. In fact, only three companies included ECs in new drug applications to the US and EU. As shown in Fig. 3, a significant number of companies are considering EC settings for future applications in EU and the US (28% and 38%, respectively) and seven companies tried to set ECs as part of PACs, suggesting that many companies are contemplating using the comparably shorter review period of a PAC submission to gain experience before deciding on the future use of ECs. As the EU’s Change Variation guidance is revised to allow the use of ECs [20] companies may leverage existing experience from other companies to determine the actual benefits of defining ECs which may also increase the use of QbD principles during drug development.

As discussed previously, before the publication of ICH Q8/9/10, new product submissions to Japan required sponsors to assign symbols or annotations indicating the post-approval change category that would be used for specific parameters. This use of Approved Matters in Japan has been in use since 2005 and is similar to the concept of ECs.

The ICH Q12 guideline has been effective in Japan since 2021 and states that “(2) The “Establishment Conditions (ECs)” is an element that is considered necessary to ensure product quality, and if a change is made, regulatory procedures are required. Since it is an approved matter, it should be stated in the manufacturing and marketing approval application as before. Additionally, changes reported as minor change notification still need to comply with the Enforcement Regulations of the Pharmaceuticals and Medical Devices Act (hereinafter referred to as the “Regulations”). Article 47, “Guidelines for Matters to be Stated in Applications for Manufacturing and Marketing Approval of Pharmaceuticals, etc. Based on the Amended Pharmaceutical Affairs Law” (Notice of the Director of the Examination and Management Division of the Pharmaceuticals and Food Bureau of the Ministry of Health, Labour and Welfare No. 0210001 dated February 10, 2005), “Entries related to the specifications and test method columns of the application for approval of pharmaceuticals, etc., and their changes” (Issued by the Pharmaceutical and Herbal Drugs Trial on July 30, 2021) No. 6, Notice of the Director of the Pharmaceutical Evaluation and Management Division, Pharmaceuticals and Public Health Bureau, Ministry of Health, Labour and Welfare). (3) The “Product Lifecycle Management (PLCM) Document” in Q12 consists of information on the EC and related change categories, the relevant post-approval CMC commitments, and the post-approval change management implementation plan (hereinafter referred to as the “PACMP”). Information on the EC and related change categories should be included in the application for approval as before.“ [14] and it is clearly stated that the manufacturing process description in the Japanese regulatory commitment is the same as ECs in Q12. As explained before, in Japan, new drug applications based on QbD development have become more common in recent years. However, many products were developed without applying QbD principles and must continue to use the process of symbols and annotations as was done before ECs were an option.

Comparing to EU and the US, Japan requires more conservative regulatory reporting for the same post approval change [21] in spite of the Notification No. 0210001 [14] which states: “The example described in this application for approval is only an example, and in the actual application for approval, the contents of the application for approval shall be in accordance with Paragraph 2.1, and the determination of the classification of changes and notable matters shall be in accordance with Paragraphs 2.2 and 2.3, and should be described on a case-by-case basis according to the characteristics of each drug.“ [14].

If a company’s PQS is robust, it is likely that there would be no difference between the change reporting category based on the traditional risk assessment approach and ECs if they had been set. In fact, this was suggested in responses from respondents in EU and the US who well-understood the concept of ECs (Fig. 7).

The post approval change process based on the change guidelines [16–19] that have been implemented in EU and the US before Q8 became effective can be applied to products developed according to the “minimal approach” defined in Q8. This method of change assessment needs resources at the time the change is planned yet can lead to an appropriate submission and review process nonetheless. This approach may be better to support post-approval changes for products not developed by a QbD approach. To avoid misreporting, regulatory authorities confirm that the reporting category is correct. In Japan, however, this approach is not possible and instead the annotation system must be applied at the time of the initial application.

Submissions to countries that do not have a fully staffed review agency often experienced long and/or unpredictable review timelines [22]establishment of ECs can make it easier to estimate workload as the regulatory reporting category is already known. Even though the change type is pre-defined, it is still necessary to provide information about the risk assessment and to generate data for implementation of the changes to facilitate appropriate PQS lifecycle management. Data requirements are not reduced when using ECs.

And as shown in Fig. 5, it is preferable for EU and the US to evaluate the risk of the changes when a change is planned instead of defining and referring to ECs even though having the ECs in place may make is easier to define a post-approval change category. The continued use of existing GMP and post-approval change guidelines remains in place and is used regularly [16–19].

In Japan, even before the use of Q12 tools such as ECs were defined, companies were required to use annotations explaining future change reporting categories. These categories set after PMDA’s review were more conservative compared to the change categories in the EU and US guidelines. FDA explains challenges and opportunities for applying Q12 to existing products based on their Established Conditions Pilot experience that ECs were not a consideration at the time of process development and regulatory approval. However, developing and evaluating EC proposals for products developed pre–ICH Q8 (i.e. without formal criticality assessments for process parameters) may be achieved by capturing and leveraging extensive manufacturing experience [23].

As discussed before, the use of ECs in EU and the US has not yet become widespread. The application of Q12 ECs varies by region, even among Japan, EU and the US. It is anticipated that achieving a common understanding globally will take considerable time.

Taking these factors into account, it is considered effective to address the discrepancies in procedures across the three regions by introducing and implementing a post-approval change guideline in Japan similar to those in place in the EU and the US in addition to the traditional method of setting and operating symbols/annotations for future change categories.

## Conclusion

Our survey was conducted between April and June 2024 and this paper was written in Feb 2025. During this period, a new notification [15] was issued in Japan on September 30 that allows risk-based changes similar to the concept of those in EU and the US change guideline-based PACs. However, these guidelines have not yet been published. In addition, the EU is in the process of revising their variations guidance based upon an upcoming change in legislation that will allow for the use of ECs [20].

It is expected that the use of ECs in the EU and the US will start with limited cases, and PACs by existing change guidelines will continue to be carried out in parallel considering the data and discussion. Therefore, a regulatory commitment in the EU and the US will include both ECs and the traditional approach sometimes having both for the same product.

In Japan, in addition to the traditional PACs system based on the annotations that are the same concept as ECs, it is necessary to proceed with the development of guidelines equivalent to EU and US change guidelines [16–19] This will allow for similar conditions among the EU, US and Japan and thus the same level of risk-based PACs even in a hybrid regulatory commitment.

To help prevent and address drug shortage situations, it will be helpful to set a defined review timeline across regulatory authorities for post-approval changes as well as the document and data expectations to ensure timely approval of these changes.

## Data Availability

Data is provided within the manuscript.
